# Does Tai Chi relieve fatigue? A systematic review and meta-analysis of randomized controlled trials

**DOI:** 10.1371/journal.pone.0174872

**Published:** 2017-04-05

**Authors:** Yu Xiang, Liming Lu, Xiankun Chen, Zehuai Wen

**Affiliations:** 1 The Second Clinical College, Guangzhou University of Chinese Medicine, Guangzhou, Guangdong, China; 2 Key Unit of Methodology in Clinical Research, Guangdong Provincial Hospital of Chinese Medicine, Guangzhou, Guangdong, China; 3 National Center for Design Measurement and Evaluation in Clinical Research, Guangzhou University of Chinese Medicine, Guangzhou, Guangdong, China; University of Edinburgh, UNITED KINGDOM

## Abstract

**Background:**

Fatigue is not only a familiar symptom in our daily lives, but also a common ailment that affects all of our bodily systems. Several randomized controlled trials (RCTs) have proven Tai Chi to be beneficial for patients suffering from fatigue, however conclusive evidence is still lacking. A systematic review and meta-analysis was performed on all RCTs reporting the effects of Tai Chi for fatigue.

**Methods:**

In the end of April 2016, seven electronic databases were searched for RCTs involving Tai Chi for fatigue. The search terms mainly included Tai Chi, Tai-ji, Taiji, fatigue, tiredness, weary, weak, and the search was conducted without language restrictions. Methodological quality was assessed using the Cochrane Risk of Bias tool. RevMan 5.3 software was used for meta-analysis. Publication bias was estimated with a funnel plot and Egger’s test. We also assessed the quality of evidence with the GRADE system.

**Results:**

Ten trials (n = 689) were included, and there was a high risk of bias in the blinding. Two trials were determined to have had low methodological quality. Tai Chi was found to have improved fatigue more than conventional therapy (standardized mean difference (SMD): -0.45, 95% confidence interval (CI): -0.70, -0.20) overall, and have positive effects in cancer-related fatigue (SMD:-0.38, 95% CI: -0.65, -0.11). Tai Chi was also more effective on vitality (SMD: 0.63, 95% CI: 0.20, 1.07), sleep (SMD: -0.32, 95% CI: -0.61, -0.04) and depression (SMD: -0.58, 95% CI: -1.04, -0.11). However, no significant difference was found in multiple sclerosis-related fatigue (SMD: -0.77, 95% CI: -1.76, 0.22) and age-related fatigue (SMD: -0.77, 95% CI: -1.78, 0.24). No adverse events were reported among the included studies. The quality of evidence was moderate in the GRADE system.

**Conclusions:**

The results suggest that Tai Chi could be an effective alternative and /or complementary approach to existing therapies for people with fatigue. However, the quality of the evidence was only moderate and may have the potential for bias. There is still absence of adverse events data to evaluate the safety of Tai Chi. Further multi-center RCTs with large sample sizes and high methodological quality, especially carefully blinded design, should be conducted in future research.

**Registration number:**

PROSPERO CRD42016033066

## Background

Although no one can exactly quantify or document fatigue [1], fatigue is a common symptom not only deeply related to most acute and chronic diseases, but also to everyday life. It is not only common, but problematic, for people with conditions such as cancer, multiple sclerosis, and rheumatoid arthritis [2]. The National Comprehensive Cancer Network (NCCN) defined cancer related fatigue as ‘an persistent, unusual, subjective feeling of tiredness correlated with cancer or cancer treatment that obstruct to normal functioning’ [3]. Definition of fatigue was also described as “a subjective feeling of lacking mental and/or physical energy, which was perceived by the caregiver or individuals interfering with usual and desired activities” [4]. Because of its subjective nature, fatigue can only be gauged by self-reported or caregiver-reported questionnaires [5]. Fatigue generally lasts longer than somnolence [6]. Tiredness is a state of temporary decreasing in strength and energy, which may be experienced as a partial of fatigue [7]. Some authors simply divided fatigue into acute and chronic fatigue [2]. Acute fatigue occurs in healthy populations, with a rapid onset and short duration. After a period of rest and exercise, it is generally relieved. Chronic fatigue mainly affects clinically disordered individuals and is onset gradually, persists and develops over time. It usually can’t be alleviated by usual recovery techniques [6]. As a symptom, fatigue is a common complaint among most people, and many ailments are accompanied by fatigue. However, it is often ignored, under-diagnosed, and seen as a natural result of physical deterioration [8].

A previous study had shown that 10.6% of women and 10.2% of men complained of fatigue for ≥ 1 month in the South London general practice attenders [9]. The prevalence rate of chronic fatigue was 10.7% in general Chinese population [10]. Among older adults with myocardial infarction, fatigue is frequently reported to be one of the most serious barriers to physical activity [11]. Fatigue occurs in 50%-83% of patients with multiple sclerosis [12]. Among breast cancer patients 58%-94% undergoing treatment and 56%-95% who are post-chemotherapy experience fatigue [13]. Although the methods, standards, and results of these studies are not always consistent, it is undeniable that fatigue is a common symptom from which many patients suffer.

The mechanisms behind fatigue are unclear [5], however they may be related to a patient’s physical condition. There is no panacea for fatigue other than treating the symptoms [5]. Evidence has shown that exercise including walking, running, jogging, swimming, resistance (strengthening) training, stretching, aerobic exercise can counter fatigue among sufferers of chronic fatigue syndrome [14], multiple sclerosis [15], fibromyalgia [16] and among cancer survivors [17,18]. So we supposes that Tai Chi, a traditional Chinese martial art, may be an effective treatment for patients suffering from fatigue.

Tai Chi has popular in China for several centuries. Many different types of Tai Chi exist, but most consist of movement, meditation and breathing, while concentrating on the mind and maintaining low intensity [19, 20], and further modulate various aspects of the body including the physical, the psychological, mood and spirit [21]. In the theory of Chinese medicine (CM), Tai Chi can maintain the harmony between *qi* and the *blood*, keep *yin* and *yang* in balance and also enhance immunity [22, 23]. These properties are both important in relieving fatigue and maintaining energy. Q*i*, the energy which promotes the body’s movement, can circulate around the entire body freely if *yin* and *yang* are kept in balance [23].

Tai Chi may relieve fatigue via different mechanisms of action. Firstly, through slow movement and weight shifting, Tai Chi may relieve stress, make people more happy [24] and promote relaxation [25]. Secondly, the proven efficacy of Tai Chi to enhance aerobic capacity and immune function [26] and to improve pain [27], depression and psychological well-being [28] may be beneficial to relieve fatigue.

An advantage of Tai Chi is that it is easy to learn, teach, and popularize, and more reports on evidence of its effects should lead to it becoming even more popular. As a low impact exercise, Tai Chi may be ideal for people with fatigue, lack of exercise or who do not have active lifestyles [19]. Several studies have reported that Tai Chi plays a critical role in fighting fatigue [29–32]. However, there not been explicit studies to reach a conclusion on Tai Chi’s effects on fatigue. Others have shown no difference between Tai Chi groups and control groups [33,34]. In addition, most of the studies focus on only one ailment [32,35,36]. As far as we know, the majority of the literature on Tai Chi intervention for fatigue is empirical, and uses small sample sizes. Few of the existing studies have explored fatigue as the primary outcome. To date, there have been no systematic reviews nor meta-analyses to evaluate the effects of Tai Chi for fatigue, but single RCTs based on a specific population in a certain place. This systematic review evaluates the effects and safety of Tai Chi for fatigue, and provides an overall understanding of the current situation, as well as problems in this field.

## Methods

This review has been registered in the PROSPERO database (PROSPERO Register code: CRD42016033066, http://www.crd.york.ac.uk/PROSPERO/), and reported according to the PRISMA Statement (S1 PRISMA Checklist) [37].

### Eligibility criteria

Randomized controlled trials (RCTs) including parallel, crossover-design or wait listed were included in this review without any language or literature quality restrictions. Only the first phase of trial will be included for the crossover-design RCTs. Participants were individuals with fatigue, regardless of any other factors such as age, sex, or current health. The intervention group included those using Tai Chi alone or Tai Chi combined with conventional medication. The control group included those using other forms of physical activity either with or without conventional treatment, conventional treatment only, placebo, or no treatment at all. The control group did not use Tai Chi [38]. There were no limitations to intervention and follow up times.

The primary outcome in this study was fatigue, estimated by a questionnaire. Fatigue scales (such as the Fatigue Severity Scale, the Fatigue Symptom Inventory, the Fatigue Scale of Motor and Cognitive Functions, and the Multidimensional Fatigue Symptom Inventory) and fatigue subscales as partials of other scales (such as the quality of life scale) represent the most detailed fatigue-related data available. Secondary outcomes included: (1) depression assessed using the Beck Depression inventory or other self-reported scales of depression; (2) sleep assessed using the Self-Rating Scale of Sleep or the Pittsburgh Sleep Quality index; (3) vitality assessed using the vitality subscale of the Medical Outcome Study Short Form 36 or the Multidimensional Fatigue Symptom Inventory-Short Form Vigor subscale scores.

### Information sources and search strategies

Seven databases were searched electronically including the China National Knowledge Infrastructure (CNKI) (from 1979), the Chinese Biomedical Medical (CBM) Database (from 1978), the Chinese Scientific Journals Database (VIP) (from 1964), Wanfang (from 1990), the Cochrane Library (from 1989), PubMed (from 1966), and Ovid EMBASE (from 1980). The search terms included Tai Chi, Taijiquan, Tai Chi Chuan, fatigue, tired (see also S1 Appendix). We also searched Clinical Trials.gov for unpublished clinical trials, as well as other websites and references to uncover other sources. The search deadline was April 30, 2016, and the search strategy, as described S1 Appendix, varied based on the character of each database.

### Selection of studies

With EndNote X6.0 software, two reviewers (Yu Xiang and Liming Lu) removed duplicates independently, and then eliminated obviously unrelated studies by screening titles and abstracts. Next, we independently screened the remaining titles and abstracts according to the inclusion criteria. In this step, we excluded any ineligible studies, and recorded the reasons for their exclusion. If based on the title and abstract, we still could not determine whether an article fit our criteria, we screened the full text. For studies that either potentially or definitely fit our criteria, the full paper was retrieved.

Discrepancies between the two reviewers were settled by discussion. If the two reviewers could not reach an agreement, a third reviewer (Zehuai Wen) was asked to moderate. Multiple published versions of the same trial were either treated as a single study, or the most comprehensive one was chosen.

### Data extraction and data items

A data collection form designed with EpiData 3.1 software (ver. 270108, the EpiData Association) was used to extract data on the publications. The form was first used in two studies, and then amended if needed, before complete information was extracted. We contacted authors to obtain any data that was missing in the original publications. The following data was extracted and input into EpiData: (1) general information (title, author, publication year, journal, country); (2) characteristics of participants (age, diagnosis of participants, stage of condition); (3) study design and number of participants; (4) intervention and control (type, frequency, duration); and (5) outcomes and measurement tools.

### Risk of bias assessment

Two authors (Yu Xiang and Liming Lu) independently judged the risk of bias assessment [39] for each study as either low, high, or unclear risk of bias, with discrepancies settled through discussion, with another review author (Zehuai Wen) mediating if necessary. Seven domains of the risk of bias assessment tool [39] were assessed including: (1) random sequence generation; (2) allocation concealment; (3) blinding of the participants and personnel; (4) blinding of outcome assessment; (5) incomplete outcome data; (6) selective reporting bias; (7) other bias.

### Data analysis

We used RevMan 5.3 software developed by the Cochrane Collaboration to conduct statistical analyses. The primary outcome was fatigue measured by either fatigue scales or fatigue subscales. Depression, vitality, and sleep were generally reported as continuous data. If scales or subscales were consistent, we calculated mean differences (MD) between the two groups with 95% confidence intervals (CI). If scales or subscales were inconsistent across trials, the outcomes were synthesized with standardized mean difference (SMD) and its 95% CI. A chi-square test was used to perform heterogeneity analysis, and *I*^*2*^ was used to estimate heterogeneity size. If *I*^*2*^ was < 50% and p-value was > 0.1, we chose the fixed-effects model. If *I*^*2*^ was ≥ 50% and the p-value was < 0.1, this showed that statistical heterogeneity existed among studies. If this situation was without clinical heterogeneity, a random effect model would be used. If the pooled results had clinical heterogeneity, the subgroup analysis would be performed based on patients characteristics, control interventions and the outcome measurement tools were adopted to handle this issue, as well as sensitivity analysis if necessary.

If the number of included studies was greater than or equal to 10, we used a funnel plot to assess the publication bias. Egger’s test was be used if the number of included studies was less than 10, or if it was difficult to estimate publication bias via funnel plot. Because funnel plot is just a figure, different people have different opinions. Subjective error would occurred easily. If a dot located in the remote area of the funnel plot, we were not sure if there was publication bias.

To retrieve missing data, we e-mailed study authors in hope they would reply. We allowed three months for their responses.

### Data synthesis

Data could be pooled if it was clinically significant and suitable for pooling. If not, we conducted a narrative synthesis of the data. For three-arm studies, we chose the low-impact exercise or the conventional treatment as the control group. If at least two studies included the same outcome, pooled analysis of the outcomes was performed.

We undertook subgroup analysis based on different conditions, different comparison interventions, different intervention length, different frequency, different duration and different measurement tools. This was done if more than two RCTs were included for each condition.

We performed sensitivity analysis based on four aspects: low quality, sample size, and age-related fatigue unrelated to the condition. This was done to assess outcome stability.

### Assessment of quality levels of evidences

We assessed the quality of evidence body for our systematic review using the Cochrane Collaboration Network GRADE (the Grading of Recommendations Assessment Development and Evaluation) [40]. Our assessment consisted of two parts: (1) We made an overall assessment on Tai Chi for fatigue without taking any reasons into consideration; (2) we assessed the quality of evidence according to different conditions related to fatigue. A summary of findings table and a GRADE evidence profile was created online with GRADEpro software at http://www.guidelinedevelopment.org/.

## Results

### Study selection

**Fig 1:**
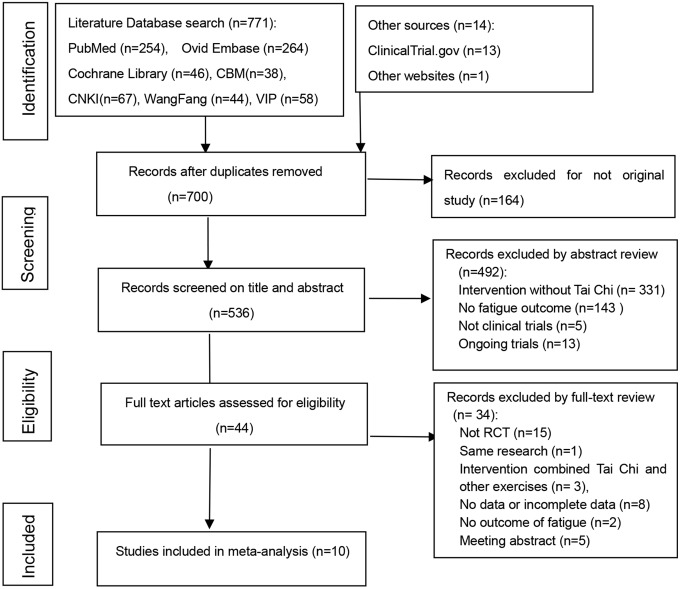
Flow diagram of study selection and identification.

There were 785 citations left after literature searches, and 700 after duplicates removed. After screening the titles and abstracts, we were left with 44 studies. We then retrieved their full-texts. Ten studies [41–50] (n = 689) met the inclusion criteria, in which all were parallel controll design, with three Chinese articles and seven English articles. Fig 1 presented details of the PRISMA flow diagram.

In total, 34 studies were excluded. Fig 1 presents the details and reasons for exclusion, most of which are categorized into the following six domains. 1) The study design was not relevant to RCT (n = 15); 2) They had replicated study (n = 1); 3) They involved unconventonal interventions including yoga, acupuncture and Chaihu Shugan power in addition to Tai Chi as main therapies in the research (n = 3); 4) There was insufficient data on fatigue (n = 8); 5) There was no outcome of fatigue (n = 2); 6) Conference abstracts (n = 5). Of the excluded 8 studies which there were insufficient data, two described fatigue outcome in words without data, one provided only median, four provided data that cannot be used to calculate SMD. One study we didn’t get full-text. For these studies, we sent e-mails to the authors, two authors had replied to us. One author sent the full text to us that we didn’t find before, but the data was useless. Both of the two authors said the original data has been destroyed. While others did not reply within three months.

### Study characteristics

A total of 689 participants with fatigue in the 10 trials were included. Nine trials [41–47, 49, 50] were conducted in single centers while one study [48] was conducted in five centers. The settings of the included studies were different, and included China, the United States, Germany and Spain. Four trials [43, 46–48] followed-up after intervention to assess the long-term effects of Tai Chi, while the others only evaluated immediately following intervention. The durations of follow-up included 3 months [43, 46], 6 months [48], and 16 months [50].

The conditions in the included studies consisted of cancer [41–43], multiple sclerosis [44, 45], rheumatoid arthritis [46], chronic and primary insomnia [47], chronic obstructive pulmonary disease (COPD) [48] and age-related fatigue [49, 50], and were in stabilized stages. The interventions in the control group, which included conventional therapy [41, 44], low-impact exercise [42], sham Qigong [43], relaxation exercises [45], stretching and wellness education [46], sleep seminars [47], no intervention [48, 50], and fast walking [49], could be regarded as conventional treatment or relaxation exercise. Tables 1 and 2 provide detailed information on the study characteristics.

**Table 1:** Characteristics of participants in the included studies.

studies	location	Number of centers	Number of patients	Age (years)	Characteristics and stages of condition
Jiang MY 2013 [41]	China: Shanghai	1	30/30	46~75	Advanced lung cancer Lung cancer
Zhang LL 2016 [42]	China: Taizhou	1	38/36	62.8	undergoing 2–4 21-day cycles of cisplatin-based chemotherapy (ECOG PS) 0–3
Larkey LK 2015 [43]	US: Arizona	1	42/45	40~75	Stages 0-III breast cancer; 6 months to 5 years past primary treatment
Burschka JM 2014 [44]	Germany: Bayreuth	115/17	15/17	42.6/43.6	Any multiple sclerosis type, ability to walk without a walking aid, EDSS score <5, and relapse-free for the past 4 weeks
Castro-Sánchez AM 2012 [45]	Spain: Granada	1	36/35	18~75	Multiple sclerosis, VAS pain score >4 for at least 2 months, EDSS≤7.5
Wang CC 2008 [46]	US: Boston	1	10/10	≥18	Functional class I and II rheumatoid arthritis
Irwin MR 2014 [47]	US: Los Angeles	1	48/25	> 55	Chronic and primary insomnia
Chan AW 2013 [48]	China: Hong Kong	5	70/67	55~88	COPD and were ambulatory
Lin Li 2012 [49]	China: Hengyang	1	31/33	> 60	Fatigue without serious ailments
Li GP 2011 [50]	China: Hunan	1	38/36	> 50	Fatigue without serious ailments

**Note**: Studies are listed by lead author and publication year. Age is stated as mean and/or range. Number of patients is shown as the number of patients in the intervention and control groups.

**Table 2:** Characteristics of included studies.

Study	Randomizing method	Generation of random sequences	Blinding	Baseline reports	Intervention		Outcomes	Outcome Measurement tools
					Experimental group	Control group		
Jiang MY 2013 [41]	Random	No description	Not mentioned	Balanced	Yang-style 24-form Length = 30 days Duration = 30 min Frequency = Twice a day	Conventional nursing Length = 30 days	Fatigue Sleep	BFI SRSS
Zhang LL 2016 [42]	Random	Computer generated random number	Not mentioned	Balanced	Simplified Yang style 5 to 10 minutes of warm-up, followed by Tai Chi Length = 12 weeks Duration = one hour Frequency = every other day Began on the tenth day of 21-day chemotherapy cycle, 4 cycles in total.	Low-impact exercise intervention Length = 12 weeks Duration = one hour Frequency = every other day. Began on tenth day of 21-day chemotherapy cycle, 4 cycles in total.	Fatigue Vitality	MFSI-SF, MFSI-SF vigor subscale
Larkey LK 2015 [43]	Stratified random	No description	Blinded to patients and outcome assessment	Balanced	Qigong/Tai Chi (Easy) 1^st^ to 2^nd^ week: Sessions, 60min long, twice a week. 3^rd^ to 12^th^ week: Sessions, 60min long, once a week. In-home Tai Chi: at least 30 minutes per day, 5 days per week. Length = 12 weeks	Sham Qigong (Placebo control group) 1^st^ to 2^nd^ week: Sessions, 60min long, twice a week. 3^rd^ to 12^th^ week: Sessions, 60min long, once a week. Home practicing: at least 30 minutes per day, 5 days per week. Length = 12 weeks	Fatigue Sleep Depression	FSI, Pittsburgh Sleep Quality Index, Beck Depression Inventory
Burschka JM 2014 [44]	Blocked assignment	No description	Not mentioned	Balanced	Yang-style 10-form, Duration = 90 minutes Frequency = weekly sessions. (1^st^ month: 4–5 times per session. Following 5 months: 6–8 times per session.) Length = 6 months	Conventional therapy Length = 6 months	Fatigue Depression	FSMC CES-D
Castro-Sánc hez AM 2012 [45]	Random	Computer generated Random table	Blinded to researchers, patients and outcome assesors	Balanced	Ai-Chi exercise Length = 20 weeks Duration = 60 minutes Frequency = twice a week Beginning and ending with 10 minutes of relaxation exercise.	Relaxation exercise group Length = 20 weeks Duration = 60 minutes Frequency = twice a week	Fatigue Depression	FSS, Beck Depression Inventory II
Wang CC 2008 [46]	Random	Computer generated random number	Not mentioned	Balanced	Classical Yang style Session: Duration = 60 minutes Frequency = twice a week. In-home Tai Chi: at least 20 minutes per day Length = 12 weeks	Stretching and wellness education Session: Duration = 60 minutes (including 40 minutes of education and 20 minutes of stretching) Frequency = twice a week Home exercise: at least 20min strength exercises once a day. Length = 12 weeks	Fatigue Depression Vitality	VAS, CES-D, Vitality of SF-36
Irwin MR 2014 [47]	Random	Computerized random number generator	Binded to outcome assessors	Balanced	Tai Chi Chih (TCC) Length = 4 months	Sleep seminar education control (SS) Length = 4 months	Fatigue Sleep Depressive	MFSI, Epworth Sleepiness Scale, IDSC-C
Chan AW 2013 [48]	Random	Computer generated randomizer	Blinded to outcome assessors	Balanced	Tai chi Qigong Length = 3 months Duration = one hour Frequency = twice a week.	Usual medical treatment (usual care) Length = 3 months	Fatigue	Borg scale
Li L 2012 [49]	Random	No description	Not mentioned	Balanced	Tai Chi Length = 6 months Duration = 40~60 minutes Frequency = 5 times per week	Fast walking Length = 6 months Duration = 40~60 minutes Frequency = 5 times per week, 90~120 steps per minute.	Fatigue Depression Vitality	Simplified POMS
Li GP 2011 [50]	Blocked assignment	No description	Not mentioned	Balanced	24 form Tai Chi 1 months: teaching and learning 2 months: practicing. 1 hour every weekday 2.5 months: strenthening practice. Length = 5.5 months	Way of life remained unchanged Length = 5.5 months	Fatigue Depression Vitality	POMS-SF

**Note**: BFI: Brief Fatigue Inventory; MFSI-SF: Multidimensional Fatigue Symptom Inventory-Short Form; FSI: Fatigue Symptom Inventory; FSMC: Fatigue Scale of Motor and Cognitive Functions; FSS: Fatigue Severity Scale; VAS: Visual Analogue Scale. SRSS: Self-Rating Scale of Sleep; CES-D: Center for Epidemiological Studies Depression Scale; IDS-C: Inventory of Depressive Symptomatology. POMS: Profile of Mood States. vitality of SF-36: vitality subscale of the Medical Outcome Study Short Form 36.

Low-impact exercises: arm, neck, and leg circles, followed by stretches for upper and lower body muscle groups, along with deep abdominal breathing. Relaxation exercises: abdominal breathing with simultaneous contraction-relaxation exercises of muscle groups in the hands, arms, shoulders, face, neck, thighs, legs, and feet while standing in shoulder-depth water.

### Risk of bias assessment

**Fig 2:**
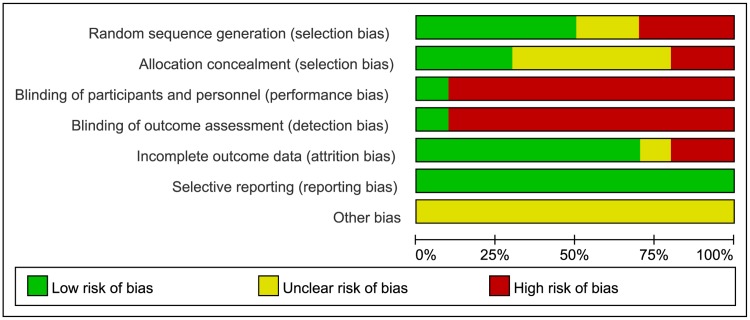
Risk of bias graph: the reviewers’ judgments about each risk of bias item presented as percentages across all included studies.

We assessed the risk of bias in the included studies based on the recommendations in the methods section of the Cochrane Handbook 5.1.0 [39]. The detailed quality assessment for the ten included studies is showed in Table 3 and Fig 2.

**Table 3:** Methodological quality assessment of included studies.

Studies	Random sequence generation (selection bias)	Allocation concealment (selection bias)	Blinding		Incomplete Outcome data (attrition bias)	Selective reporting (reporting bias)	Other bias
			Participants, personnel	Assessors			
Jiang MY 2013 [41]	U	U	H	H	L	L	U
Zhang LL 2016 [42]	L	U	H	H	H	L	U
Larkey LK 2015 [43]	H	U	L	L	L	L	U
Burschka JM 2014 [44]	H	H	H	H	L	L	U
Castro-Sánchez AM 2012 [45]	L	L	H	H	L	L	U
Wang CC 2008 [46]	L	L	H	H	U	L	U
Irwin MR 2014 [47]	L	L	H	H	L	L	U
Chan AW 2013 [48]	L	U	H	H	L	L	U
Li L 2012 [49]	U	U	H	H	H	L	**U**
Li GP 2011 [50]	H	H	H	H	L	L	U

**Notes**: U, unclear risk of bias; L, low risk of bias; H, high risk of bias

Five studies [42, 45–48] reported the random method of using a computer-generated random sequence, while one study [50] used only a random number table to divide communities into experimental community and control community. Two studies [41, 49] lacked descriptions of the method of random sequence generation. One study [43] used stratified randomization based on 2 factors, and another [44] used block assignment which was produced according to which patient came on the day of Tai Chi courses. Seven studies [41–44, 48–50] did not describe how allocation concealment was conducted while one study [47] stated that researchers, including assessors and participants, were not allowed near the randomization list. The randomization list was managed by an individual who was not in contact with participants and researchers. Two studies [45, 46] used sealed opaque envelopes to perform allocation concealment.

Eight studies [41, 42, 44, 46–50] lacked details on whether participants and administrators were blinded, however it was clear blinding had been broken due to obvious differences between intervention group and control group. Although one study [45] mentioned it was double-blind, we also determined the blind had been broken because of disparities between the two groups. Six trials [41, 42, 44, 46, 49, 50] did not employ blinding of the outcome assessments, One study [42] said that it was impossible to blind the participants or data collector. After contacted authors, one study [44] replied to us that they didn’t blind to outcome assessors and data collectors. One study [49] didn’t reply to us. Three studies [41, 46, 50] didn’t leave e-mail and phone numbers. Three studies [45, 47, 48] were performed blind, but with blinding easily broken. Only one study [43] successfully blinded participants, researchers and outcome assessors for consistent comparisons between the Qigong/Tai Chi Easy and Sham Qigong groups.

Three studies [41, 44, 45] reported all patients’ outcomes, while one study [46] failed to mention whether there was missing data. Two studies [42, 49] had a high dropout rate and provided detailed explanations, but did not do specific statistical analysis. Three studies [43, 47, 50] reported low dropout rates within the range of statistical estimations provided in advance of the studies. One study [48] had a high drop-out rate, but it had occurred at random, and intention-to-treat analysis had been performed. All studies reported all outcomes the demonstrated by the methods sections. The information necessary for judging the risk of other bias of all studies was insufficient.

### Synthesis of results

#### Primary outcome

Although participants of the ten trials [41–50] were afflicted by different conditions and the scales were different, all trials focused on the validity of Tai Chi in treating symptoms of fatigue. Their goals and the methods of measurements were consistent. Therefore, we conducted pooled analysis. SMD and 95% CI were adopted in evaluating the effect of Tai Chi on fatigue, based on various scales.

**Fig 3:**
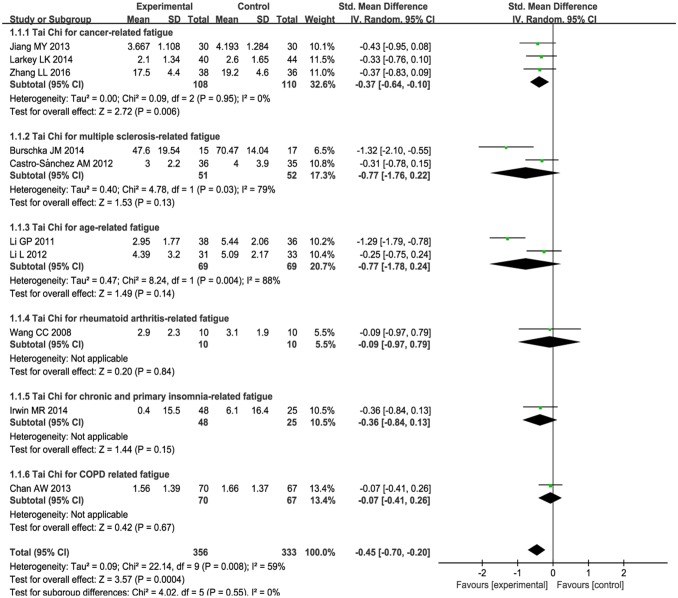
Meta-analysis of Tai Chi for fatigue. A random effect model was performed to test for high statistical heterogeneity. Subgroup analysis was based on three different conditions including cancer, multiple sclerosis and age-related fatigue. Only descriptive analysis was performed for Tai Chi for rheumatoid arthritis, primary insomnia and COPD related fatigue.

Ten studies [41–50] reported that fatigue symptoms subsided after Tai Chi intervention. A random effect model was used, because the statistical heterogeneity of the pooled results was significant (*P* = 0.008, *I*^2^ = 59%). The fatigue score was reduced more in the Tai Chi group post-intervention than it was in the control group (SMD: -0.45, 95% CI: -0.70, -0.20, *P* = 0.0004) (see Fig 3).

#### Subgroup analysis

**Fig 4:**
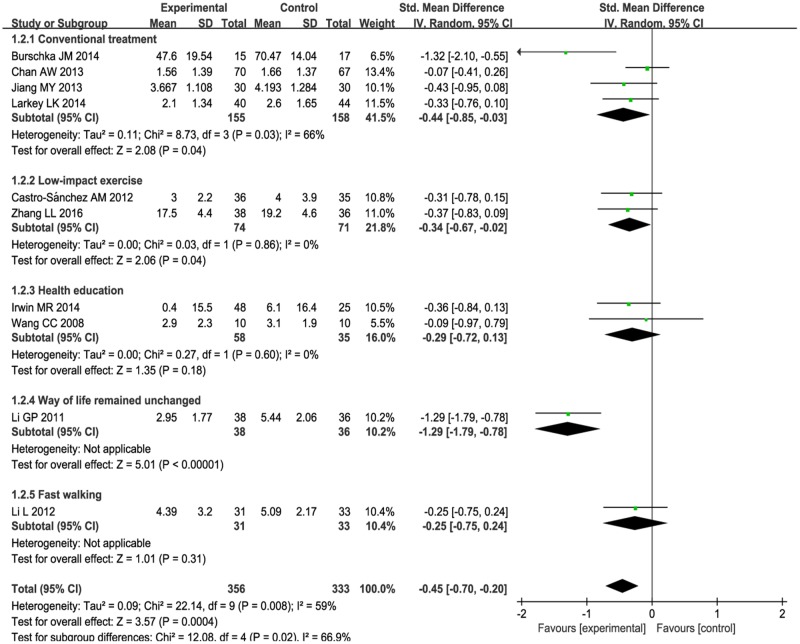
Meta-analysis of Tai Chi for fatigue. Subgroup analysis was based on two different control groups including conventional treatment, low-impact exercise and health education. A random model was performed to test for high statistical heterogeneity. Only descriptive analysis was performed for Tai Chi compared with fast walking, and the way of life remained unchanged.

**Fig 5:**
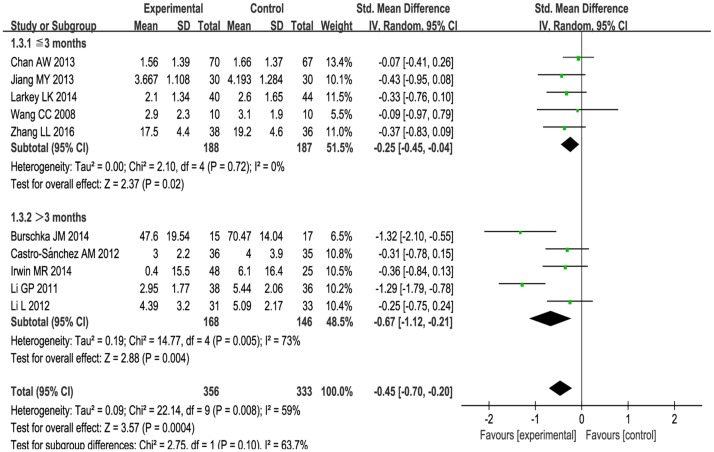
Forest plot of the subgroup analysis of Tai Chi for fatigue based on intervention length.

**Fig 6:**
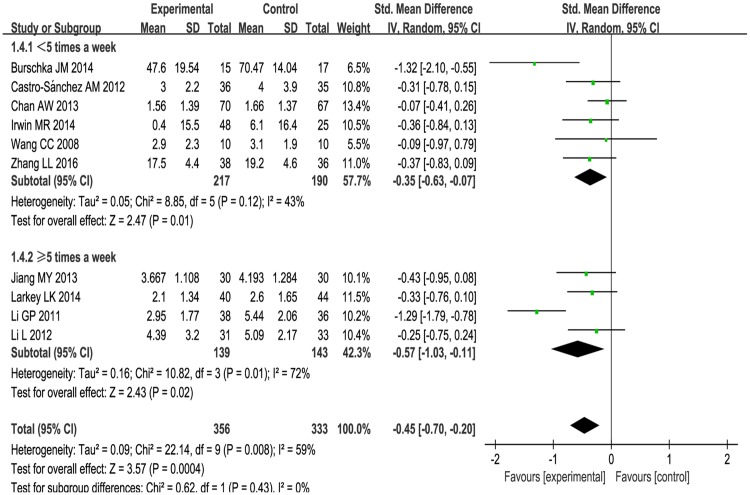
Forest plot of the subgroup analysis of Tai Chi for fatigue based on different frequency.

**Fig 7:**
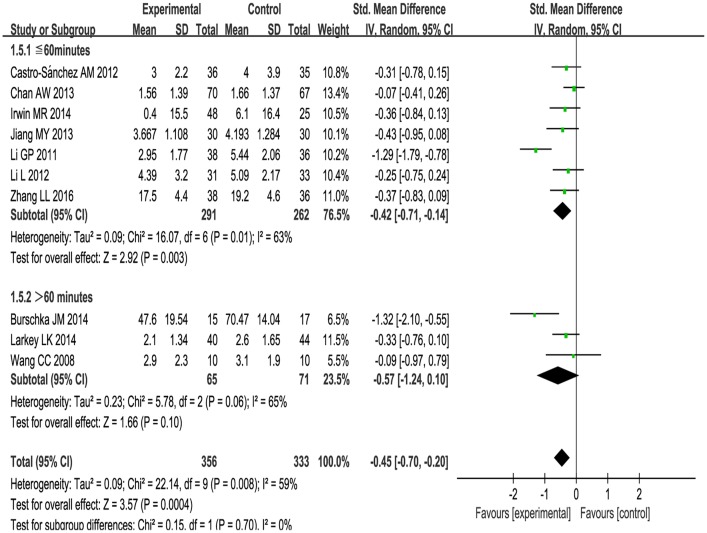
Forest plot of the subgroup analysis of Tai Chi for fatigue based on different duration.

1) Subgroup analysis was performed according to different ailments in the included studies. This included:
  (1) The effects of Tai Chi for cancer-related fatigue:
    Cancer-related fatigue was presented in three studies [41–43], and the pooled effect was statistically significant (SMD: -0.38; 95% CI: -0.65, -0.11; *P* = 0.006) (see Fig 3), which showed that Tai Chi intervention significantly improved cancer-related fatigue.
  (2) The effects of Tai Chi for multiple sclerosis-related fatigue in two studies [44,45]:
    There was no significance (SMD: -0.77; 95% CI: -1.76, 0.22; *P* = 0.13) (see Fig 3) due to high heterogeneity (*P* = 0.03, *I*^2^ = 79%).
  (3) Tai Chi for age-related fatigue in two studies [49,50]:
    There was no significance (SMD: -0.77; 95% CIL: -1.78, 0.24; *P* = 0.14) with high heterogeneity (*P* = 0.004, *I*^2^ = 88%) (see Fig 3).
  For rheumatoid arthritis-related fatigue, one study [46] reported no difference between Tai Chi group and stretching and wellness education (mean changes, 2.9 versus 3.1, *P* > 0.05). For chronic and primary insomnia-related fatigue, Irwin MR et al.[47] reported that Tai Chi improved fatigue greater compared with sleep seminar education control (mean changes, 0.4 versus 6.1, *P* < 0.05). One study [48] reported no changes in fatigue level for COPD patients between Tai Chi and usual medical treatment (mean changes, 1.56 versus 1.66, *P* > 0.05). These three studies [46–48] can only be described and not statistically analyzed, because there was only one study in its subgroup.
2) Subgroup analysis was conducted based on different comparison interventions in the included studies.
  (1) The aggregated results indicated that Tai Chi improved fatigue greater than conventional treatment control [41,43,44,48] (SMD:-0.44, 95%CI:-0.85, -0.03) but with high heterogeneity (*I*^2^ = 66%, *P* = 0.03) (See Fig 4) and low-impact exercise control [42, 45] (SMD:-0.34, 95%CI:-0.67, -0.02) with heterogeneity was low (*I*^2^ = 0%, *P* = 0.98) (See Fig 4).
  (2) Comparing with health education [46,47], no significant differences were observed between two groups (SMD:-0.29, 95%CI: -0.72, 0.13). The heterogeneity was low (*I*^2^ = 0%, *P* = 0.60) (see Fig 4).
  (3) One study reported that Tai Chi improved fatigue greater than fast walking control (mean changes, 4.39 versus 5.09, *P* < 0.05) [49]. One study reported that Tai Chi relived fatigue more comparing with the way of life remained unchanged (mean changes, 2.95 versus 5.44, *P* < 0.05) [50]. These two studies [49, 50] can only be described and not statistically analyzed, because there was only one study in its subgroup.
3) The subgroup analysis was performed based on different intervention length:≤ 3 months, > 3 months. For length≤ 3months, Tai Chi significantly improved fatigue (SMD: -0.25, 95%CI: -0.45, -0.04, *P* = 0.02) with low heterogeneity (*I*^2^ = 0%, *P* = 0.72) [41–43, 46, 48] (See Fig 5). For length > 3 months, Tai Chi significantly reduced fatigue (SMD: -0.67, 95%CI: -1.12, -0.21, *P* = 0.004) with high heterogeneity (*I*^2^ = 73%, *P* = 0.005) [44,45,47, 49,50] (see Fig 5).
4) The subgroup analysis based on frequency: < 5 times a week, ≥ 5 times a week. For < 5 times a week, significantly improved fatigue in Tai Chi group (SMD: -0.35, 95%CI: -0.63, -0.07) with heterogeneity (*I*^2^ = 43%, *P* = 0.12) [42, 44–48](see Fig 6). For ≥ 5 times a week, Tai Chi was more effective in relieving fatigue (SMD: -0.57, 95%CI: -1.03, -0.11) with heterogeneity (*I*^2^ = 72%, *P* = 0.01) [41, 43, 49, 50] (see Fig 6).
5) The subgroup analysis based on different duration:≤ 60minutes, > 60minutes. For ≤ 60minutes, Tai Chi improved fatigue greater than control group (SMD: -0.42, 95%CI: -0.71, -0.14) with high heterogeneity (*I*^2^ = 63%, *P* = 0.01) [41,42,45, 47–50] (see Fig 7). For > 60 minutes, Tai Chi significantly improved fatigue (SMD: -0.57, 95%CI: -1.24, 0.10) with high heterogeneity (*I*^2^ = 65%, *P* = 0.06) [43,44, 46] (see Fig 7).

#### Sensitivity analysis

**Fig 8:**
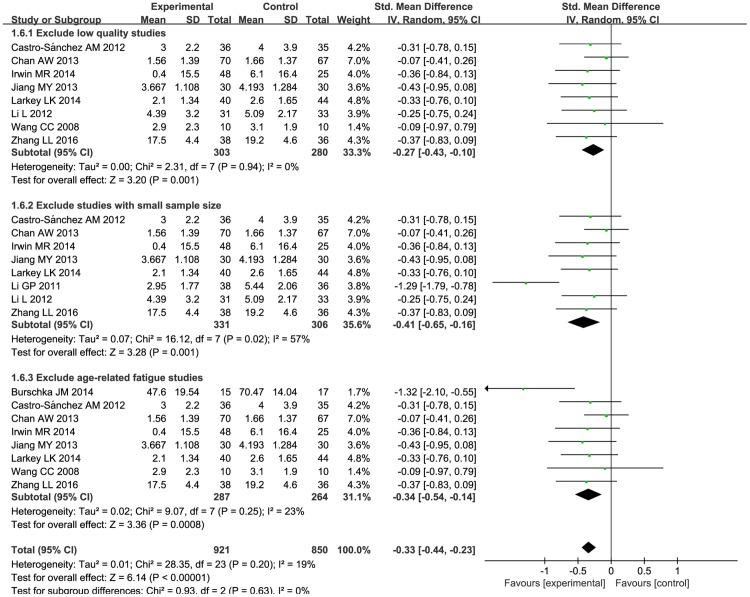
Forest plot of the sensitivity analysis of Tai Chi for fatigue. Sensitivity analysis was performed based on excluding studies of low quality, those with small sample sizes, and those in which fatigue was due to age, not a particular condition. A random effect model was performed to manage the high heterogeneity.

Sensitivity analysis was performed based on excluding studies of low quality, small sample size, and those in which age-related fatigue was not due to a particular condition. First, we excluded two studies of low quality [44, 50]. After this step, statistical heterogeneity disappeared. The pooled effects showed that there was a difference between the Tai Chi group and the conventional group (SMD: -0.27; 95% CI: -0.43, -0.10; *P* = 0.001) (Fig 8). Next, we rejected two studies [44, 46] with small sample sizes. At this point, there was a difference between two groups (SMD: -0.41; 95% CI: -0.65, -0.16; *P* = 0.001) (Fig 8). Finally, we eliminated two studies [49,50] on fatigue that was related to age not a particular condition. After this, statistical significance was found (SMD: -0.34; 95% CI: -0.54, -0.14; *P* = 0.0008) (Fig 8).

The result had no significant change after using 3 different sensitivity analyses. This proved that the result was stable and reliable.

#### Secondary outcomes

*Vitality* Vitality was reported in four studies [42, 46, 49, 50]. The increase in the vitality score of the Tai Chi group was greater than that of the conventional treatment group (SMD: 0.63; 95% CI: 0.20, 1.07; *P* = 0.004) (S1 Fig). Heterogeneity (*P* = 0.06, *I*^2^ = 59%) (S1 Fig) was significant for the low quality of some studies. We still can conclude that Tai Chi was beneficial to patient’s vitality.

*Sleep* Sleep was also reported in three studies [41, 43, 47]. Sleep improvement among the Tai Chi group was greater than it was in the conventional treatment group (SMD: -0.32; 95% CI: -0.61, -0.04; *P* = 0.03) (S2 Fig). The heterogeneity was no significant (*I*^2^ = 0%, *P* = 0.50) (S2 Fig).

*Depression* Depression was reported in 7 studies [43–47, 49,50]. Among of them, 6 trials [44–47,49,50] showed that Tai Chi can improve depression, and one trial [43] showed no significance between two groups. The pooled effect in meta-analysis showed a significant difference between the Tai Chi group and the conventional treatment group (SMD: -0.58; 95% CI: -1.04, -0.11; *P* = 0.01) with high heterogeneity (*P*<0.0001, *I*^2^ = 80%) (S3 Fig) for the low quality of some studies. It can be concluded that Tai Chi is beneficial for depression.

#### Adverse events

Eight studies [41–44, 47–50] did not report adverse events. Two studies [45, 46] reported that there were no adverse events.

#### Publication bias

**Fig 9:**
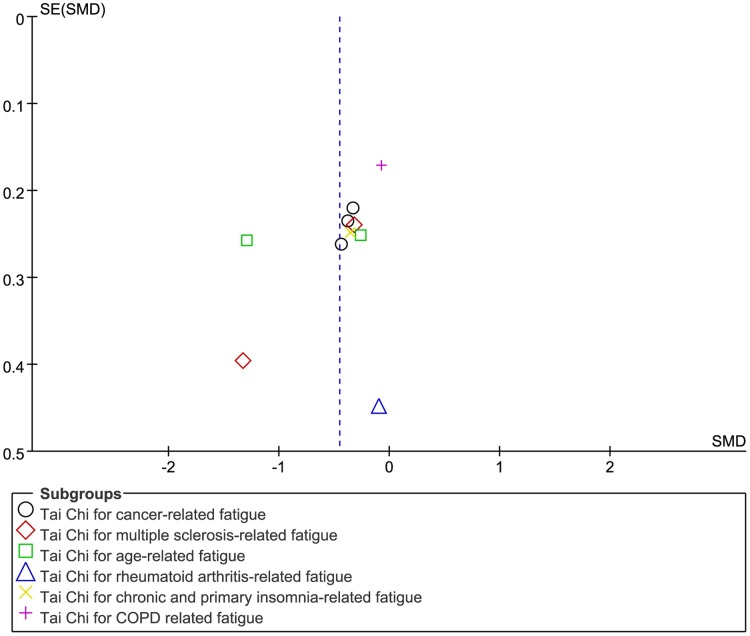
Funnel plot of publication bias of all included trials comparing Tai Chi exercise with control interventions.

A funnel plot was drawn, and it was difficult to assess publication bias (Fig 9). However, no significant publication bias was indicated after performing Egger's test (P = 0.178) (Table 4).

**Table 4:** Egger’s test.

Std_Eff	Coef	SE	t	p	95% CI
Slop	0.305	0.497	0.61	0.557	-0.842, 1.451
Bias	-2.972	2.012	-1.48	0.178	-7.612, 1.669

**Notes**: Std_Eff: Standard Effect; Coef.: Coefficient; SE: Standard Error

### Quality levels of evidences

The level of evidence quality was assessed with the GRADE system. First, we made an overall assessment on Tai Chi for fatigue. The result showed that the quality of evidence was moderate, because most of the studies lacked detailed descriptions of blinding. The detailed information and explanation are shown in the GRADE evidence profile (S2 Appendix) and the summary of finding table (S3 Appendix). In the next step, the quality of evidence according to fatigue related to different ailments was assessed separately. This was done because after subgroup analysis there were wide difference between Tai Chi for different conditions related to fatigue (Fig 3). The results showed that the quality of evidence was moderate in Tai Chi for cancer-related fatigue because of the poor design of blinding, and very low in multiple sclerosis-related fatigue and age-related fatigue because of the low methodological quality, high heterogeneity and small sample size (S2 and S3 Appendixes). For the studies of Tai Chi for rheumatoid arthritis-related fatigue, chronic and primary insomnia-related fatigue and COPD related fatigue, evidence quality was low because of the small sample size and low methodological quality (S2 and S3 Appendixes).

## Discussion

### Summary of findings

The ten included studies proved that Tai Chi was beneficial in relieving fatigue. This systematical review and meta-analysis showed that improvement of fatigue symptoms was greater among Tai Chi groups than control interventions but with high heterogeneity. However, after excluding the two studies of lowest methodological quality [45, 50], we found that heterogeneity disappeared and the effect was still greater in Tai Chi group. We also performed sensitivity analyses with considering other two factors to prove the stability and reliability of the results. This included sample size and age-related fatigue unrelated to particular conditions, which showed that the results were stable and reliable.

Considering the subgroup analysis, the results showed that Tai Chi intervention significantly improved cancer-related fatigue with low heterogeneity, while there was no significant effect for multiple sclerosis or age-related fatigue with high heterogeneity. For rheumatoid arthritis and COPD patients, only one RCT ([46, 48], respectively) reported no difference between Tai Chi group and control group (stretching and wellness education, usual medical treatment, respectively). For chronic and primary insomnia-related fatigue, Irwin MR et al. [47] reported that Tai Chi improved fatigue greater compared with sleep seminar education control.

Tai Chi improved fatigue greater than conventional treatment and low-impact exercise control. While comparing with health education, no significant difference was found between two groups. The pooled results showed that the duration time of practicing Tai Chi ≤ 60minutes was better than > 60 minutes, and no difference in the length of ≤ 3months and > 3months and the frequency of < 5 times a week and ≥ 5 times a week.

This review also showed that Tai Chi was more effective in treating sleep difficulty, lack of vitality and depression. For the sleep difficulty, a previous systematic review and meta-analysis [51] showed that Tai Chi exercise was beneficial to improve self-rated sleep quality for elderly people. In this study, three trials [41, 43, 47] reported the sleep outcome and the heterogeneity of the pooled results was not significant. For depressive symptoms, seven studies [43–47, 49, 50] reported this outcome. Although the result showed Tai Chi was more effective, the heterogeneity was high. In addition, a previous systematic review and meta-analysis [52] showed that Tai Chi was no significant effect for depression. Deeper research was required. For vitality, the heterogeneity of the pooled result was significant.

The quality of the included studies was not high, with five studies [41, 43, 44, 49, 50] lacking description of the randomization method, and seven studies [41–44, 49, 50] not mentioning allocation concealment. Selection bias was existed. Only one study [43] was successfully blinded to researchers, participants, and outcome assessors.

Eight studies [41–44, 47–50] did not report adverse events. Two studies [45, 46] reported that there were no adverse events. Four trials [43, 46–48] performed follow-up, with the most time-intensive one [47] having a 16 month follow-up period. Reports on adverse effects of Tai Chi are mosly centered on joint damage or muscle and ligament injury caused by exercising with too much force and poor postures [53]. These are primarily isolated to these specific problems, but still cannot be ignored. There is still absence of adverse events data, further investigation of Tai Chi should be conducted.

### Findings in relation to previous studies and reviews

To our knowledge, this study is the first review based on RCTs assessing the efficacy and safety of Tai Chi for people suffering from fatigue. To date, there have been numerous reviews of exercise such as Tai Chi, Yoga, walking, jogging, and running as a treatment for fatigue related to a variety of conditions. *BMC Cancer* published a review [54] on supervised exercise for cancer-related fatigue including aerobic exercise and resistance training. Another review [55] which explored aquatic exercise for fibromyalgia also addressed fatigue. In another review [56], fatigue was also an outcome. Our review included only one type of exercise for sufferers of fatigue, Tai Chi. Thus, our study did not include comprehensive evidence on a wide variety of exercise. However, our results showed that Tai Chi improves fatigue, and this was consistent with the effects of exercise intervention.

### Limitations

The varying degrees of fatigue of patients with different ailments who were included in this review may have significantly affected our results. Due to the limited number of eligible trials, we did not restrict participants to certain demographics or conditions. Moreover, the small-scale trials limited performance of subgroup analysis. The type of Tai Chi intervention, the length, duration and frequency of intervention and outcome measure tools varied, but we could not make detailed subgroups due to the limited number of included studies. This is a limitation in our review. This may have influenced the explanatory effect and the soundness of pooled effects. Fatigue was measured by validated scale in our included studies. Meanwhile, most of included studies treated fatigue as secondary outcome. So, many of them lack of detailed description of the fatigue. Due to the limited number of included studies, we can’t limited the severity of fatigue in the inclusion criteria. After systematic literature search, we knew that the present situation of the research on Tai Chi for fatigue is just in an initial and exploratory stage. We will update our review if there are new studies.

Although SMD was used to present the fatigue outcome in the meta-analysis, the pooled results may also be affected by different outcome measurement tools in the included studies. However, SMD could be understand as a pooled effect size, for example, SMD = -0.45, could be interpreted as the decrease of score between experimental group and control group achieved 45% of the pooled standard deviation.

Heterogeneity among studies was significant, which may be explained by the low methodological quality. This is because heterogeneity disappeared after excluding low quality studies. Sample size also has an impact on the effects of Tai Chi for fatigue.

There was high risk of bias in the blinding. Although it was difficult to blind investigators and participants, outcome assessors should have been blinded in order to avoid expectation bias. There was selection bias because of the poor randomization method in some studies. Attrition bias was also existed. Two studies [42, 49] did not conduct statistical analysis due to incomplete outcome data. Another two studies [44, 46] had sample sizes that were too small.

### Implications for clinical practice

Firstly, we summarized the current condition of Tai Chi for fatigue and provided information to support a future clinical trial. It is convenient for other researchers to do further research. Secondly, we made an overall assessment of Tai Chi for fatigue. The pooled effect of Tai Chi was greater than conventional therapy and low-impact exercise (general daily activities). We didn’t focus on specific population. So, the conclusion was suited to all fatigued people. Thirdly, we provide the overall quality of evidence by the GRADE system to users, which is convenient for them to use and popularize the results.

## Conclusions

The overall aggregated result showed that Tai Chi achieved better gains in relieving fatigue compared to the control interventions. For the subgroup analysis, Tai Chi was more beneficial for cancer-related fatigue. However, for multiple sclerosis-related fatigue, age-related fatigue, there were no significant difference between two groups. Tai Chi improved fatigue greater than conventional treatment and low-impact exercise control, while no difference was observed comparing with health education control. The length between ≤ 3 months and > 3 months and the frequency between < 5 times a week and ≥ 5 times a week, the pooled results indicated that they all have significant difference. However, in the duration≤ 60 minutes, Tai Chi was improved fatigue greater. In the duration > 60 minutes, there were no difference between two groups. So, the duration≤ 60 minutes may be better than > 60 minutes.

Although existing preliminary evidence has shown that Tai Chi can alleviate fatigue, the overall quality of these studies has been low. The GRADE Working Group grades of the evidence for Tai Chi’s effect on fatigue were moderate level of quality, meaning that the true effect is likely close to the estimate of the effect. However, there is a possibility that it is substantially different. Therefore, clinical practitioners should treat this evidence with caution when making decisions. The GRADE quality of evidence in Tai Chi for cancer related-fatigue was moderate, while for other ailments the evidence was low or very low. Tai Chi may be better for cancer patients with fatigue than for patients with other conditions. However, we cannot exclude the influence of the small sample sizes and low methodological quality in the existing studies of Tai Chi for fatigue related to other ailments. Tai Chi also achieved better gains in sleep difficulty, lack of vitality and depression. There is still absence of evidence, further safety investigation of Tai Chi should be undertaken. Additional multi-center RCTs with large sample sizes and high methodological quality are needed, especially those with careful blinding. This will lead to further understanding of Tai Chi’s effects in treating fatigue.

## Supporting information

S1 PRISMA Checklist (DOC)

S1 Appendix Search strategy. (DOC)

S2 Appendix The GRADE evidence profile. (DOC)

S3 Appendix GRADE Summary of Findings table. (DOC)

S1 Fig Meta-analysis of Tai Chi for vitality. (TIF)

S2 Fig Meta-analysis of Tai Chi for sleep. (TIF)

S3 Fig Meta-analysis of Tai Chi for depression. (TIF)
