# Supplementary material for: Does Tai Chi relieve fatigue? A systematic review and meta-analysis of randomized controlled trials
Source: PLoS One. 2017 Apr 5;12(4):e0174872. doi: 10.1371/journal.pone.0174872 (PMC5381792; doi:10.1371/journal.pone.0174872)
Supplement: S1 Appendix — (DOC) [file pone.0174872.s002.doc]

S1 Appendix: Search Strategy

**Key words:**

English: Tai-ji, Taiji, Tai Chi, Tai Ji Quan, Chi,Tai, Taichi,Taijiquan,

Tai Chi Chuan, Ji Quan,Tai, Quan,Tai Ji, tai-yi, shadow boxing

Fatigue, lassitude, Asthenia, Asthenias, muscle weakness,

Muscle weaknesses, Chronic fatigue syndrome, CFS, fatigue syndrome myalgicncephalomyelitis, Exhaustion, exhaust，exhausted，tire，tired, burnout(倦怠)，worn out，wear out，give out， tiredness，weary，weak，feeble，lacking in strength，royal free disease，muscular weakness，adynamia

clinical trials

Chinese：太极，太极拳

疲劳，疲乏,劳累，乏力，无力，虚弱，肌无力，慢性疲劳综合征，疲劳综合征，疲倦，精疲力竭，倦怠,筋疲力尽，衰弱，疲惫，困倦，体力缺乏，肌肉无力，肌力下降，无精打采，精神萎靡，无生气。

随机，临床试验

**Search terms:**

**CNKI**

(SU = '太极' OR SU = '太极拳') AND ( SU = '疲劳' OR SU = '疲乏' OR SU = '乏力' OR SU = '肌无力' OR SU = '慢性疲劳综合征' OR SU = '疲劳综合征' OR SU = '慢性疲劳症候群' OR SU = '劳累' OR SU = '疲倦' OR SU = '精疲力竭' OR SU = '倦怠' OR SU = '筋疲力尽' OR SU = '衰弱' OR SU = '疲惫' OR SU = '困倦' OR SU = '体力缺乏' OR SU = '肌肉无力' OR SU = '肌力下降' OR SU = '无精打采' OR SU = '精神萎靡' OR SU = '无生气') AND (FT = '随机' OR FT = '临床试验')

**Wangfang database**

(太极 or 太极拳) and (疲劳 or 疲乏 or 乏力 or 肌无力 or 慢性疲劳综合征 or 疲劳综合征or 疲劳综合征群 or 劳累 or 疲倦 or 精疲力竭 or 倦怠 or 筋疲力尽 OR 衰弱 OR 疲惫OR 困倦 OR 体力缺乏 OR 肌肉无力 OR 肌力下降 OR 无精打采 OR 精神萎靡 OR 无生气)

**VIP**

(M=太极+M=太极拳)*(M=疲劳+M=疲乏+M=乏力+M=肌无力+M=慢性疲劳综合征+M=疲劳综合征+M=疲劳综合征群+M=劳累+M=疲倦+M=精疲力竭+M=倦怠+M=筋疲力尽+M=衰弱+M=疲惫+M=困倦+M=体力缺乏+M=肌肉无力+M=肌力下降+M=无精打采+M=精神萎靡+M=无生气)

**CBM**

#1=太极

#2=太极拳

#3=慢性疲劳综合征

#4=疲劳综合征

#5=疲劳

#6=疲乏

#7=乏力

#8=肌无力

#9=劳累

#10=疲倦

#11=精疲力竭

#12=倦怠

#13=筋疲力尽

#14=衰弱

#15=疲惫

#16=困倦

#17=体力缺乏

#18=肌肉无力

#19=肌力下降

#20=无精打采

#21=精神萎靡

#22=无生气

#23= ((#1) OR (#2))

#24= ((((((((((((((((((((((((((((((((((((((#4) OR (#5))) OR (#6))) OR (#7))) OR (#1))) OR (#8))) OR (#9))) OR (#10))) OR (#11))) OR (#12))) OR (#13))) OR (#14))) OR (#15))) OR (#16))) OR (#17))) OR (#18))) OR (#19))) OR (#20))) OR (#21))) OR (#22))

#25= [((#23) AND (#24))](javascript:toDoRelimitSearch();)

**The Cochane Library**

#1 Taiji

#2 tai chi

#3 Tai Ji Quan

#4 Chi, Tai .

#5 Taichi

#6 Taijiquan

#7 Tai Chi Chuan

#8 Tai-ji

#9 t’ai chi chuan

#10 Martial Arts

#11 shadow boxing

#12 Ai Chi

#13 t’ai chi

#14 Tai Chi Quan

#15Ji Quan,Tai

#16 Quan,Tai Ji

#17 tai-yi

#18 tai ji

#19 Tai Ji

#20 taji*

#21 tai-chi

#22taichiquan

#23 #1 or #2 or #3 or #4 or #5 or #6 or #7 or #8 or #9 or #10 or #11 or #12 or #13 or #14 or #15 or #16 or #17 or #18 or #19 or #20 or #21 or #22

#24fatigue

#25tired

#26weary

#27weariness

#28exhaustion

#29exhausted

#30astenia syndrome

#31asthenic syndrome

#32(lack or loss or lost) and (energy or vigo?r)

#33apath$

#34lassitude

#35weak

#36letharg$

#37 feel and (drained or sleep$ or sluggish)

#38exhaust

#39tire

#40burnout

#41worn out

#42 give out

#43 tiredness

#44feeble

#45adynamia

#46royal free disease

#47muscular weakness

#48muscle weakness

#49chronic fatigue syndrome

#50CFS

#51fatigue syndrome

#52myalgic encephalomyelitis

#53Asthenia

**#**54 vitality

#55 lacklustre

#56 languidness

#57 languor

#58 listlessness

#59 astenia

#60asthenic

#61 #24 or #25 or #26 or #27 or #28 or #29 or #30 or #31 or #32 or #33 or #34 or #35 or #36 or #37 or #38 or #39 or #40 or #41 or #42 or #43 or #44 or #45 or #46 or #47 or #48 or #49 or #50 or #51 or #52 or #53 or #54 or #55 or #57 or #58 or #59 or #60

#62 #23 and #61

**PubMed**

((((((((((((((((((((((("tai ji"[MeSH Terms] OR ("tai"[All Fields] AND "ji"[All Fields]) OR "tai ji"[All Fields] OR "taiji"[All Fields]) OR ("tai ji"[MeSH Terms] OR ("tai"[All Fields] AND "ji"[All Fields]) OR "tai ji"[All Fields] OR ("tai"[All Fields] AND "chi"[All Fields]) OR "tai chi"[All Fields])) OR ("tai ji"[MeSH Terms] OR ("tai"[All Fields] AND "ji"[All Fields]) OR "tai ji"[All Fields] OR ("tai"[All Fields] AND "ji"[All Fields] AND "quan"[All Fields]) OR "tai ji quan"[All Fields])) OR ("tai ji"[MeSH Terms] OR ("tai"[All Fields] AND "ji"[All Fields]) OR "tai ji"[All Fields] OR ("chi"[All Fields] AND "tai"[All Fields]))) OR Taichi[All Fields]) OR ("tai ji"[MeSH Terms] OR ("tai"[All Fields] AND "ji"[All Fields]) OR "tai ji"[All Fields] OR "taijiquan"[All Fields])) OR ("tai ji"[MeSH Terms] OR ("tai"[All Fields] AND "ji"[All Fields]) OR "tai ji"[All Fields] OR ("tai"[All Fields] AND "chi"[All Fields] AND "chuan"[All Fields]) OR "tai chi chuan"[All Fields])) OR ("tai ji"[MeSH Terms] OR ("tai"[All Fields] AND "ji"[All Fields]) OR "tai ji"[All Fields])) OR (("tai ji"[MeSH Terms] OR ("tai"[All Fields] AND "ji"[All Fields]) OR "tai ji"[All Fields] OR ("t'ai"[All Fields] AND "chi"[All Fields]) OR "t'ai chi"[All Fields]) AND chuan[All Fields])) OR ("martial arts"[MeSH Terms] OR ("martial"[All Fields] AND "arts"[All Fields]) OR "martial arts"[All Fields])) OR (shadow[All Fields] AND ("boxing"[MeSH Terms] OR "boxing"[All Fields]))) OR Ai, Chi[Full Author Name]) OR ("tai ji"[MeSH Terms] OR ("tai"[All Fields] AND "ji"[All Fields]) OR "tai ji"[All Fields] OR ("t'ai"[All Fields] AND "chi"[All Fields]) OR "t'ai chi"[All Fields])) OR (("tai ji"[MeSH Terms] OR ("tai"[All Fields] AND "ji"[All Fields]) OR "tai ji"[All Fields] OR ("tai"[All Fields] AND "chi"[All Fields]) OR "tai chi"[All Fields]) AND Quan[All Fields])) OR ("tai ji"[MeSH Terms] OR ("tai"[All Fields] AND "ji"[All Fields]) OR "tai ji"[All Fields] OR ("ji"[All Fields] AND "quan"[All Fields] AND "tai"[All Fields]))) OR ("tai ji"[MeSH Terms] OR ("tai"[All Fields] AND "ji"[All Fields]) OR "tai ji"[All Fields] OR ("quan"[All Fields] AND "tai"[All Fields] AND "ji"[All Fields]))) OR tai-yi[All Fields]) OR ("tai ji"[MeSH Terms] OR ("tai"[All Fields] AND "ji"[All Fields]) OR "tai ji"[All Fields])) OR ("tai ji"[MeSH Terms] OR ("tai"[All Fields] AND "ji"[All Fields]) OR "tai ji"[All Fields])) OR (taji[All Fields] OR tajiani[All Fields] OR tajic[All Fields] OR tajick[All Fields] OR tajifei[All Fields] OR tajifi[All Fields] OR tajig[All Fields] OR tajii[All Fields] OR tajik[All Fields] OR tajika[All Fields] OR tajika28[All Fields] OR tajikana[All Fields] OR tajikawa[All Fields] OR tajikazu[All Fields] OR tajiken[All Fields] OR tajikestan[All Fields] OR tajiki[All Fields] OR ("tajikistan"[MeSH Terms] OR "tajikistan"[All Fields]) OR tajikistan's[All Fields] OR tajikistani[All Fields] OR tajiko[All Fields] OR tajiks[All Fields] OR tajikstan[All Fields] OR tajil[All Fields] OR tajima[All Fields] OR tajima'd[All Fields] OR tajima's[All Fields] OR tajima002[All Fields] OR tajimab[All Fields] OR tajimao[All Fields] OR tajimas[All Fields] OR tajimas's[All Fields] OR tajimash[All Fields] OR tajimaya[All Fields] OR tajime[All Fields] OR tajimi[All Fields] OR tajin[All Fields] OR tajinda[All Fields] OR tajinder[All Fields] OR tajine[All Fields] OR tajinere[All Fields] OR tajini[All Fields] OR tajino[All Fields] OR tajir[All Fields] OR tajiran[All Fields] OR tajiri[All Fields] OR tajirian[All Fields] OR tajiriji[All Fields] OR tajirika[All Fields] OR tajirim[All Fields] OR tajirit[All Fields] OR tajirita[All Fields] OR tajiriy[All Fields] OR tajirl[All Fields] OR tajirou2[All Fields] OR tajish[All Fields] OR tajitsu[All Fields] OR tajixanthone[All Fields] OR tajiyeva[All Fields])) OR ("tai ji"[MeSH Terms] OR ("tai"[All Fields] AND "ji"[All Fields]) OR "tai ji"[All Fields] OR ("tai"[All Fields] AND "chi"[All Fields]) OR "tai chi"[All Fields])) OR taichiquan[All Fields]) AND (((((((((((((((((((((((((((((((((((((("fatigue"[MeSH Terms] OR "fatigue"[All Fields]) OR tried[All Fields]) OR weary[All Fields]) OR ("fatigue"[MeSH Terms] OR "fatigue"[All Fields] OR "weariness"[All Fields])) OR exhaustion[All Fields]) OR exhausted[All Fields]) OR (("asthenia"[MeSH Terms] OR "asthenia"[All Fields]) AND ("syndrome"[MeSH Terms] OR "syndrome"[All Fields]))) OR (("asthenia"[MeSH Terms] OR "asthenia"[All Fields] OR "asthenic"[All Fields]) AND ("syndrome"[MeSH Terms] OR "syndrome"[All Fields]))) OR ((lack[All Fields] OR loss[All Fields] OR lost[All Fields]) AND (("Energy (Oxf)"[Journal] OR "energy"[All Fields]) OR vigo?r[All Fields]))) OR ("apathy"[MeSH Terms] OR "apathy"[All Fields])) OR ("fatigue"[MeSH Terms] OR "fatigue"[All Fields] OR "lassitude"[All Fields])) OR ("asthenia"[MeSH Terms] OR "asthenia"[All Fields] OR "weak"[All Fields])) OR ("lethargy"[MeSH Terms] OR "lethargy"[All Fields])) OR (("emotions"[MeSH Terms] OR "emotions"[All Fields] OR "feel"[All Fields]) AND (drained[All Fields] OR ("sleep"[MeSH Terms] OR "sleep"[All Fields]) OR sluggish[All Fields]))) OR exhaust[All Fields]) OR tire[All Fields]) OR burnout[All Fields]) OR (worn[All Fields] AND out[All Fields])) OR (give[All Fields] AND out[All Fields])) OR ("fatigue"[MeSH Terms] OR "fatigue"[All Fields] OR "tiredness"[All Fields])) OR feeble[All Fields]) OR adynamia[All Fields]) OR ("fatigue syndrome, chronic"[MeSH Terms] OR ("fatigue"[All Fields] AND "syndrome"[All Fields] AND "chronic"[All Fields]) OR "chronic fatigue syndrome"[All Fields] OR ("royal"[All Fields] AND "free"[All Fields] AND "disease"[All Fields]) OR "royal free disease"[All Fields])) OR ("muscle weakness"[MeSH Terms] OR ("muscle"[All Fields] AND "weakness"[All Fields]) OR "muscle weakness"[All Fields] OR ("muscular"[All Fields] AND "weakness"[All Fields]) OR "muscular weakness"[All Fields])) OR ("paresis"[MeSH Terms] OR "paresis"[All Fields] OR ("muscle"[All Fields] AND "weakness"[All Fields]) OR "muscle weakness"[All Fields] OR "muscle weakness"[MeSH Terms] OR ("muscle"[All Fields] AND "weakness"[All Fields]))) OR ("fatigue syndrome, chronic"[MeSH Terms] OR ("fatigue"[All Fields] AND "syndrome"[All Fields] AND "chronic"[All Fields]) OR "chronic fatigue syndrome"[All Fields] OR ("chronic"[All Fields] AND "fatigue"[All Fields] AND "syndrome"[All Fields]))) OR CFS[All Fields]) OR (("fatigue"[MeSH Terms] OR "fatigue"[All Fields]) AND ("syndrome"[MeSH Terms] OR "syndrome"[All Fields]))) OR ("fatigue syndrome, chronic"[MeSH Terms] OR ("fatigue"[All Fields] AND "syndrome"[All Fields] AND "chronic"[All Fields]) OR "chronic fatigue syndrome"[All Fields] OR ("myalgic"[All Fields] AND "encephalomyelitis"[All Fields]) OR "myalgic encephalomyelitis"[All Fields])) OR ("metabolism"[Subheading] OR "metabolism"[All Fields] OR "me"[All Fields])) OR ("asthenia"[MeSH Terms] OR "asthenia"[All Fields])) OR vitality[All Fields]) OR lackluster[All Fields]) OR languidness[All Fields]) OR ("cercopithecidae"[MeSH Terms] OR "cercopithecidae"[All Fields] OR "langur"[All Fields])) OR ("apathy"[MeSH Terms] OR "apathy"[All Fields] OR "listlessness"[All Fields])) OR ("asthenia"[MeSH Terms] OR "asthenia"[All Fields])) OR ("asthenia"[MeSH Terms] OR "asthenia"[All Fields] OR "asthenic"[All Fields])) AND Clinical Trial[ptyp]) AND ("1966/01/01"[PDAT] : "2016/04/30"[PDAT])

**Ovid MEDLINE /Embase**

1 tai chi/

2 taiji$.tw

3 tai chi chuan/

4 shadowboxing

5. Tai Ji/

6. Martial Arts/

7. exp Tai Ji/

8. tai chi.tw.

9. Ai Chi.tw.

10. taichi.tw.

11. tai ji.tw.

12. chi tai.tw.

14. tai-chi.tw.

15. taiji.tw.

16. exp tai chi/

17.tai-yi

18.taijiquan

19.Tai Chi Quan

20. Tai Ji Quan

21. Chi,Tai

22. Tai-ji

23. Ji Quan,Tai

24. Quan,Tai Ji

25. taji*

26. taichiquan

27. or/1-26

28. exp Fatigue/

29. fatigue$.tw.

30. (tired$ or weary or weariness or exhaustion or exhausted).tw.

31. ((astenia or asthenic) and syndrome).tw.

32. ((lack or loss or lost) adj3 (energy or vigo?r)).tw.

33. (apath$ or lassitude or weak$ or letharg$).tw.

34. (feel$ adj3 (drained or sleep$ or sluggish)).tw.

35. vitality.tw.

36.(lacklustre or languidness or languor or listlessness) .tw.

37. exhaust

38.tire

39.burnout

40worn out

41.tiredness

42.feeble

43.adynamia

44.royal free disease

45.muscular weakness

46.muscle weakness

48.chronic fatigue syndrome

49.CFS

50.fatigue syndrome

51.myalgic encephalomyelitis

52.Asthenia

53 or/28-52

54 27 and 53
