# Supplementary material for: Does Tai Chi relieve fatigue? A systematic review and meta-analysis of randomized controlled trials
Source: PLoS One. 2017 Apr 5;12(4):e0174872. doi: 10.1371/journal.pone.0174872 (PMC5381792; doi:10.1371/journal.pone.0174872)
Supplement: S2 Appendix — (DOC) [file pone.0174872.s003.doc]

S2 Appendix. The GRADE evidence profile

**Author(s)**: Yu Xiang, Liming Lu, Xiankun Chen, Zehuai Wen

**Date**: 2016/8/3

**Question**: Tai Chi compared to conventional therapy for fatigue

**Setting**: China, America, Germany, Spain

**Bibliography**: Does Tai Chi Relieve Fatigue?: A systematic Review and Meta-analysis

| **Quality assessment** | | | | | | | **№ of patients** | | **Absolute Effect (95% CI)** | **Quality** | **Importance** |
| --- | --- | --- | --- | --- | --- | --- | --- | --- | --- | --- | --- |
| **№ of studies** | **Study design** | **Risk of bias** | **Inconsistency** | **Indirectness** | **Imprecision** | **Other considerations** | **Tai Chi** | **conventional therapy** |
| Tai Chi for fatigue | | | | | | | | | | | |
| 10 | randomised trials | serious ^1^ | not serious ^2^ | not serious ^3^ | not serious | none | 356 | 333 | SMD:- **0.45**  (-0.7 , -0.2 ) | ⨁⨁⨁◯ MODERATE | IMPORTANT |
| Tai Chi for cancer-related fatigue | | | | | | | | | | | |
| 3 | randomised trials | serious ^4^ | not serious | not serious | not serious | none | 108 | 110 | SMD:- **0.37**  (-0.64, -0.1 ) | ⨁⨁⨁◯ MODERATE | IMPORTANT |
| Tai Chi for multiple sclerosis-related fatigue | | | | | | | | | | | |
| 2 | randomised trials | serious ^5^ | serious ^6^ | not serious | serious ^7^ | none | 51 | 52 | SMD:- **0.77**  (-1.76, 0.22 ) | ⨁◯◯◯ VERY LOW | IMPORTANT |
| Tai Chi for age-related fatigue | | | | | | | | | | | |
| 2 | randomised trials | serious ^8^ | serious ^9^ | not serious | serious ^10^ | none | 69 | 69 | SMD:- **0.77** (-1.78 ,0.24 ) | ⨁◯◯◯ VERY LOW | NOT IMPORTANT |
| Tai Chi for rheumatoid arthritis-related fatigue | | | | | | | | | | | |
| 1 | randomised trials | serious ^11^ | not serious | not serious | serious ^12^ | none | 10 | 10 | SMD:- **0.09** (-0.97, 0.79) | ⨁⨁◯◯ LOW | IMPORTANT |
| Tai Chi for chronic and primary insomnia-related fatigue | | | | | | | | | | | |
| 1 | randomised trials | serious ^13^ | not serious | not serious | serious ^14^ | none | 48 | 25 | SMD:- **0.36** (-0.84, 0.13 ) | ⨁⨁◯◯ LOW | IMPORTANT |
| Tai Chi for COPD related fatigue | | | | | | | | | | | |
| 1 | randomised trials | serious ^15^ | not serious | not serious | serious ^16^ | none | 70 | 67 | SMD:- **0.07**  (-0.41 ,0.26 ) | ⨁⨁◯◯ LOW | IMPORTANT |

**CI:** Confidence interval; **SMD:** Standardised mean difference

1. There were no details on blinding and the randomisation method. Although it was difficult to blind to patients and researchers, outcome assessors should be blinded.
2. The low quality of two studies led to the high heterogeneity. After excluding the two low quality studies, the heterogeneity disappeared.
3. Although there were differences between patients and the duration of intervention and follow-up, the aim of our study was to evaluate the effects of Tai Chi for fatigue, regardless of reasons.
4. Details on blinding and randomisation method were missing.
5. Details on blinding were missing. Randomisation method of one study judged to have high risk of bias.
6. There was high heterogeneity and the effect between 2 studies was different.
7. The sample size was too small, and the effect size intersected with the equivalent line.
8. Methodological quality was low.
9. There was high heterogeneity and the effect between 2 studies was different.
10. The sample size was too small, and the effect size intersected with the equivalent line.
11. Details on blinding, incomplete outcome data and other bias were missing.
12. Sample size was too small, and the effect size intersected with the equivalent line.
13. Details on blinding were missing.
14. The sample size was too small, and the effect size intersected with the equivalent line.
15. Details on blinding, allocation concealment, and other bias were missing.
16. The sample size was too small, and the effect size intersected with the equivalent line.
