# Supplementary material for: Does Tai Chi relieve fatigue? A systematic review and meta-analysis of randomized controlled trials
Source: PLoS One. 2017 Apr 5;12(4):e0174872. doi: 10.1371/journal.pone.0174872 (PMC5381792; doi:10.1371/journal.pone.0174872)
Supplement: S3 Appendix — (DOC) [file pone.0174872.s004.doc]

S3 Appendix. GRADE Summary of Findings table

| **Summary of findings:** Does Tai Chi Exercise Relieve Fatigue?: A systematic Review and Meta-analysis | | | | |
| --- | --- | --- | --- | --- |
| **Tai Chi compared to conventional therapy for fatigue** | | | | |
| **Patient or population**: fatigue  **Setting**: China, America, Germany, Spain  **Intervention**: Tai Chi  **Comparison**: conventional therapy | | | | |
| Outcomes | **Absolute Effect (95% CI)** | № of participants  (studies) | Quality of the evidence (GRADE) | Comments |
|
| Fatigue | SMD:- **0.45**  (-0.7 , -0.2 ) | 689 (10 RCTs) | ⨁⨁⨁◯ MODERATE ^1,2,3^ | 1. Details on blinding and random method were missing. Although it was difficult to blind to patients and researchers, outcome assessors should be blinded. 2. The low quality of two studies lead to the high heterogeneity. 3. Although there were differences between patients and the duration of intervention and follow-up, the aim of our study was to evaluate the effects of Tai Chi for fatigue, regardless of reason. |
| Cancer-related fatigue | SMD:- **0.37**  (-0.64, -0.1 ) | 218 (3 RCTs) | ⨁⨁⨁◯ MODERATE ^4^ | 4. Details on blinding and random method were missing. |
| Multiple sclerosis-related fatigue | SMD:- **0.77**  (-1.76, 0.22 ) | 103 (2 RCTs) | ⨁◯◯◯ VERY LOW ^5,6,7^ | 5. Details on blinding were missing. The random method of one study was judged as having a high risk of bias. 6. High heterogeneity, the effect between 2 studies was different. 7. Sample size was too small and the effect size intersected with the equivalent line. |
| Age-related fatigue | SMD:- **0.77** (-1.78 ,0.24 ) | 138 (2 RCTs) | ⨁◯◯◯ VERY LOW ^8,9,10^ | 8. Low methodological quality. 9. High heterogeneity and the effects between 2 studies were different. 10. Sample size was too small, and the effect size intersected with the equivalent line. |
| rheumatoid arthritis-related fatigue | SMD:- **0.09** (-0.97, 0.79) | 20 (1 RCT) | ⨁⨁◯◯ LOW ^11,12^ | 1. Details on blinding, incomplete outcome data and other bias were missing. 12. Sample size was too small, and the effect size intersected with the equivalent line. |
| Chronic and primary insomnia-related fatigue | SMD:- **0.36** (-0.84, 0.13 ) | 73 (1 RCT) | ⨁⨁◯◯ LOW ^13,14^ | 13. Details on blinding were missing. 14. Sample size was too small, and the effect size intersected with the equivalent line. |
| COPD-related fatigue | SMD:- **0.07**  (-0.41 ,0.26 ) | 137 (1 RCT) | ⨁⨁◯◯ LOW ^15,16^ | 15. Details on blinding, allocation concealment and other bias were missing. 16. Sample size was too small, and the effect size intersected with the equivalent line. |
| **CI:** Confidence interval; **SMD:** Standardised mean difference | | | | |
| **GRADE Working Group grades of evidence** **High quality:** We are very confident that the true effect lies close to that of the estimate of the effect. **Moderate quality:** We are moderately confident in the effect estimate: The true effect is likely close to the estimate of the effect, but there is a possibility that it is substantially different. **Low quality:** Our confidence in the effect estimate is limited: The true effect may be substantially different from the estimate of the effect. **Very low quality:** We have very little confidence in the effect estimate: The true effect is likely substantially different from the estimate of effect. | | | | |
