# Supplementary figures and images for: Does Tai Chi relieve fatigue? A systematic review and meta-analysis of randomized controlled trials
Source: PLoS One. 2017 Apr 5;12(4):e0174872. doi: 10.1371/journal.pone.0174872 (PMC5381792; doi:10.1371/journal.pone.0174872)

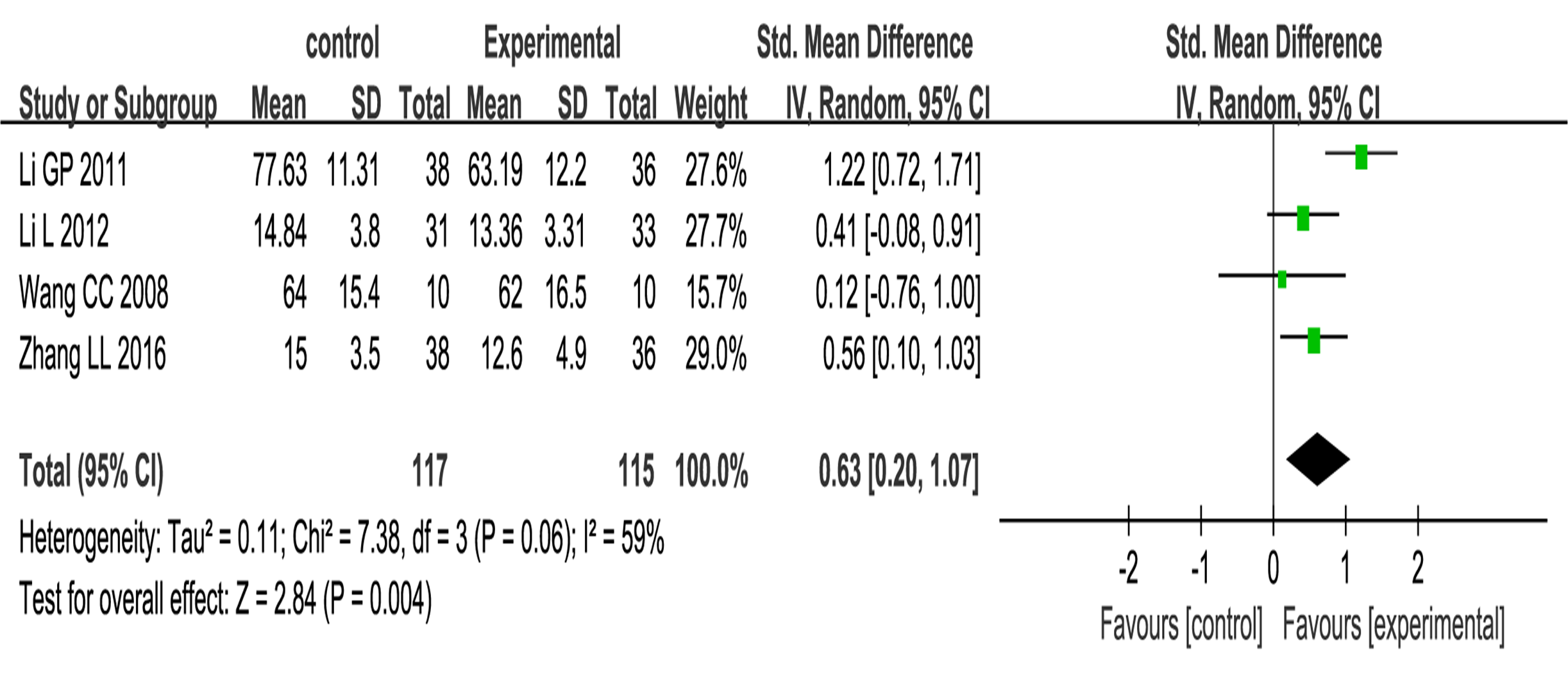

Supplement: S1 Fig — (TIF) [file pone.0174872.s005.tif]

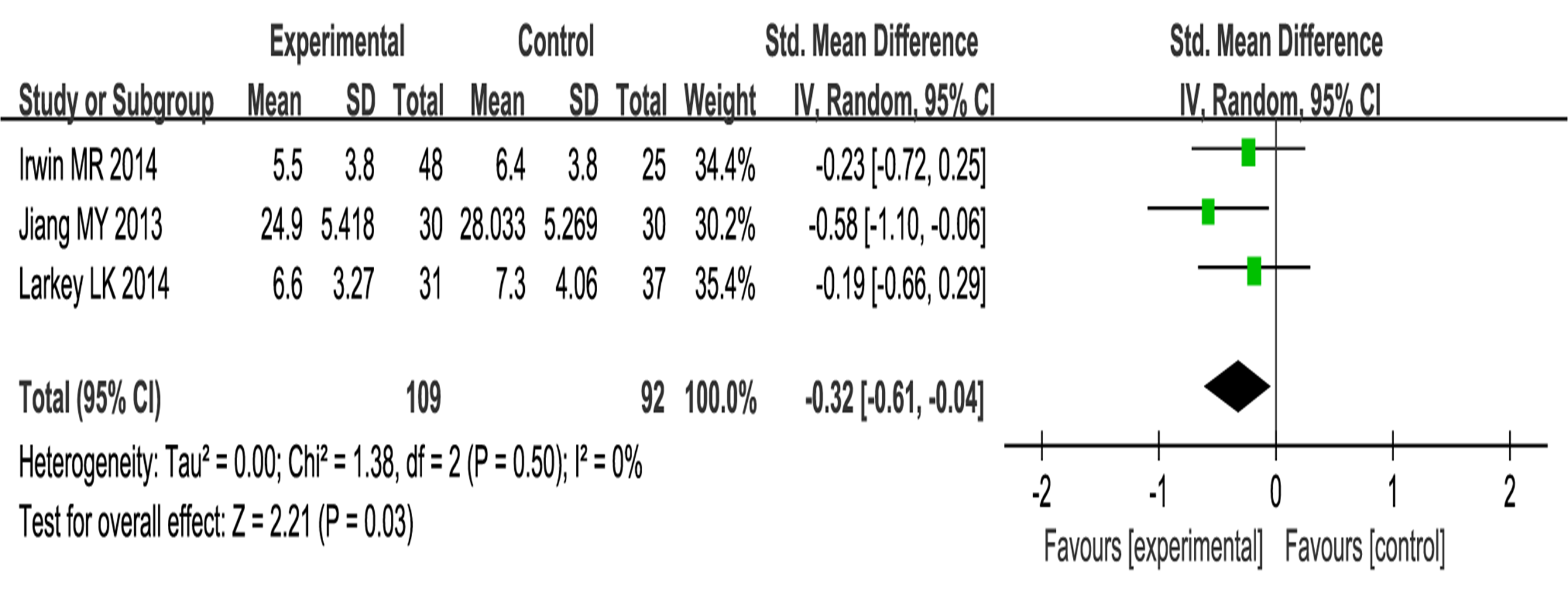

Supplement: S2 Fig — (TIF) [file pone.0174872.s006.tif]

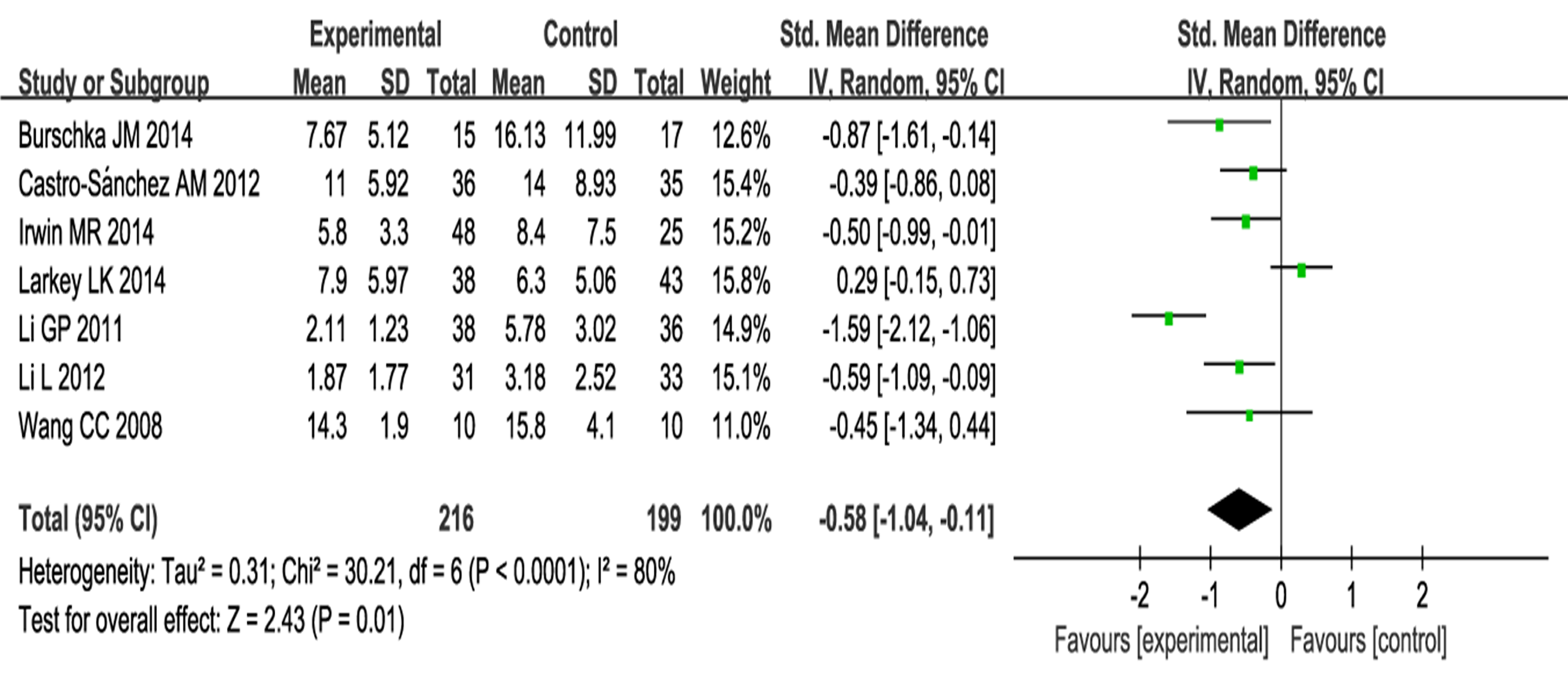

Supplement: S3 Fig — (TIF) [file pone.0174872.s007.tif]
